# Hydrophilic and Lipophilic Carbon Dots Impart Thermosensitivity to Doxorubicin Loaded Phospholipid Liposomes

**DOI:** 10.3390/ph19050668

**Published:** 2026-04-25

**Authors:** Barbara Mavroidi, Kyriaki Marina Lyra, Zili Sideratou, Dimitris Tsiourvas

**Affiliations:** Institute of Nanoscience and Nanotechnology, National Center for Scientific Research ‘‘Demokritos”, 15310 Aghia Paraskevi, Greece; b.mavroidi@inn.demokritos.gr (B.M.); k.lyra@inn.demokritos.gr (K.M.L.); z.sideratou@inn.demokritos.gr (Z.S.)

**Keywords:** carbon dots, thermosensitive liposomes, lipid bilayers, membrane permeability, doxorubicin

## Abstract

**Background/Objectives**: Hyperthermia coupled with temperature-triggered drug delivery systems, including drug-loaded thermosensitive liposomes, that exhibit increased membrane permeability at hyperthermia-relevant temperatures is a promising therapeutic strategy for cancer treatment. Our previous study revealed that nitrogen-doped carbon dots (CD) partially interact with the phospholipids of liposomes, increasing the membrane permeability of an encapsulated anticancer drug. In vitro cell experiments indicated that their presence in the culture medium, albeit at relatively high concentrations, also affect cell membrane permeability, enhancing drug internalization in cancer cells. This study aims to introduce either hydrophilic or lipophilic carbon dots into liposomes and evaluate them as thermosensitive drug delivery systems. **Methods**: Alkylated carbon dots (CD-C16) were synthesized and liposomal systems with either the lipophilic CD-C16 or the parent hydrophilic CD were prepared and efficiently loaded with doxorubicin (DOX). Following physicochemical characterization, their thermosensitivity was studied vs. time and temperature, while their effect on cell survival at 37 and 40 °C was evaluated against HEK293 and PC3 cells. **Results**: At 40 °C, for CD containing liposomes 50% DOX release is observed, whereas for CD-C16 containing liposomes 95% DOX is released within 5 min. Against PC3 cells at 40 °C, both DOX-loaded CD containing liposomes and CD-C16 containing liposomes are more potent compared to the parent drug-loaded liposomes, whereas CD-C16 containing liposomes are equally potent to free DOX. Against HEK293 cells the thermosensitive formulations at 40 °C prove even more cytotoxic, with CD-C16 containing liposomes being more potent than free DOX, but CD containing liposomes are advantageous for being less toxic than free DOX at 37 °C. **Conclusions**: Although work is needed to elucidate the mechanism at the molecular level, the results suggest that it is possible to adjust liposomal membrane permeability through the incorporation of carbon dots in order to optimize performance for hyperthermia-based applications.

## 1. Introduction

Hyperthermia entails the raising of the temperature of certain areas of the body up to above 40 °C and is being used as a therapeutic approach for various diseases over thousands of years [1,2]. Currently local hyperthermia can be achieved by employing radiofrequency, ultrasound, or microwaves and is widely employed in cancer therapeutic schemes [3,4]. Temperature increase of the tumor to 40–42 °C is typically applied together with established anti-cancer treatments, i.e., chemotherapy or radiotherapy [4]. Thermosensitisation can be successful at moderate temperatures (average temperature 41 °C), while for practical reasons minimum temperatures that can be typically achieved range between 39.5 and 40.5 °C [5]. The combination of heat with either radiation or treatment with cytostatic compounds has been proven in preclinical studies [6,7], as well as in Phase II and III clinical trials, showing improved tumor recurrence and survival in certain cases [4,5]. Our current knowledge indicates that hyperthermia leads to improved tumor blood flow, vascular permeability, and thus upregulated drug influx in the tumor, improved tumor oxygenation, or even direct cell death [3,5,8,9,10], as well as to the modulation of the immune system by activating natural killer cells and phagocytes [11,12,13,14].

It was further proposed that combining hyperthermia with drug delivery systems that can target tumors would further increase the therapeutic outcome, especially since in a number of studies it was reported that the combination of hyperthermia with a free drug does not result in an increase in drug concentration within the tumors [5]. To this end, a large number of drug delivery systems that can release their drug payload at these temperatures, and that can also target tumors either passively (by exploiting the enhanced permeation and retention effect) or actively (by the introduction of targeting groups) have been developed and investigated. Such thermally triggered drug delivery systems primarily encompass a diversity of polymeric systems, including drug-loaded polymeric micelles, polymeric nanoparticles, polymersomes, hydrogels, or polymer−drug conjugates [15,16,17,18,19,20,21], and thermosensitive, or temperature sensitive, liposomes. The ability of liposomes to release their drug load at a certain temperature is based on the property of liposomal phospholipid-based membranes to become significantly permeable at temperatures around their main lipid phase transition, as was documented in the 1970s [22,23,24]. Since then, this property has been exploited for the development of thermally triggered drug release liposomal systems, including sterically stabilized PEGylated liposomes, for anti-cancer therapy [25,26,27]. A variety of lipid formulations have been tested, with a major advance in this area being the work of Needham et al. who introduced in the typical dimyristoylphospholipid bilayer a lysophospholipid at moderate concentrations (5–10% molar), leading to liposomes exhibiting the fast release of encapsulated drugs at ca. 41 °C, while being stable (non-leaky) at 37 °C [28,29,30,31]. A large number of phospholipid formulations with additional molecules of a variety of chemical structures that induce membrane thermosensitivity have been developed and tested since then. Evidence is provided that thermosensitive liposomal formulations combined with hyperthermia maximize drug accumulation and effectiveness in tumors, as demonstrated in animal studies [5,32,33,34,35,36], while systems exhibiting the most encouraging results have led to, currently ongoing, clinical trials [37,38,39].

Our previous study on the interaction of nanoparticles with biomembranes [40] indicated that hydrophilic nitrogen-doped carbon dots (CD), that are slightly positively charged due to the presence of amino groups in their surface, interact electrostatically in a concentration-dependent manner with the slightly negatively charged membrane of liposomes, which is a close artificial analogue of a typical cell surface. This interaction was primarily manifested by a substantial increase in the bilayer permeability, especially at relatively high CD concentrations in the medium, inducing the fast release of liposomal encapsulated doxorubicin (DOX). Physicochemical analysis suggested that the carbon dots are partially embedded within the phospholipid bilayer, while in vitro cell experiments also established that the presence of free CD in the culture medium, at, however, relatively high concentrations, selectively enhanced breast cancer cell internalization of doxorubicin. This resulted in increasing its cytotoxicity, which suggests that carbon dots can, acting in a similar manner, affect cell membrane permeability as well. These results triggered the initiative to develop lipophilic functionalized CD that would more preferably reside within the bilayer and could thus modify membrane permeability at much lower concentrations. It was envisaged that this property could be further exploited for the preparation of a new class of thermosensitive liposomes employing benign carbon nanodots.

To this end, palmitoyl-functionalized carbon dots (CD-C16) were synthesized and employed for the development of PEGylated liposomes, aiming for a thermally triggered drug release system at a hyperthermia-relevant temperature. Liposomal systems containing either the lipophilic CD-C16 or the parent hydrophilic CD were prepared and efficiently loaded with doxorubicin. The chemotherapeutic drug doxorubicin hydrochloride (DOX) was chosen due to its extensive use as an anticancer agent, as well as due to its fluorescent properties that facilitate quantification and cell internalization. The effect of CD and CD-C16 on the physicochemical properties, stability, and bilayer permeability of DOX-loaded liposomes was studied as a function of time and temperature. The biological activity of carbon dots containing liposomes on cell survival at normal (37 °C) and hyperthermia-related (40 °C) temperatures was assessed in in vitro cultures of embryonic kidney-derived HEK293 cells, a non-cancerous DOX sensitive cell line [41], and in human prostate PC3 cells, a cancerous DOX-resistant cell line [42].

## 2. Results and Discussion

### 2.1. Synthesis and Physicochemical Characterization of Alkylated Nitrogen-Doped Carbon Dots (CD-C16)

Nitrogen-doped carbon dots (CD) were prepared by microwave irradiation of a citric acid/ethylenediamine aqueous solution, following the procedure described in detail in our previous publication [43]. Under the employed conditions, ethylenediamine and citric acid undergo condensation polymerization reactions, as well as dehydration, cyclization, and pyrolysis reactions. As a result, carbon nanoparticles are derived, which are composed of compact carbon cores, of oligomeric structures with various degrees of polymerization due to amide and ester groups formation, and of carboxylic, hydroxyl, and amino functional groups that are located at their surface shell [44,45,46]. The obtained CD had 1.2 ± 0.02 mmol of primary amino groups per gram at their surface, and a quasi-spherical morphology with a narrow size distribution of a mean size of 4.5 nm, while their z-potential values are close to neutral (2.5 ± 1.2 mV) [43,47] due to the presence of both carboxylic and amino groups. They also exhibit strong fluorescent properties in water after excitation in the UV region, with a quantum yield of 48% [43].

**Scheme 1:**
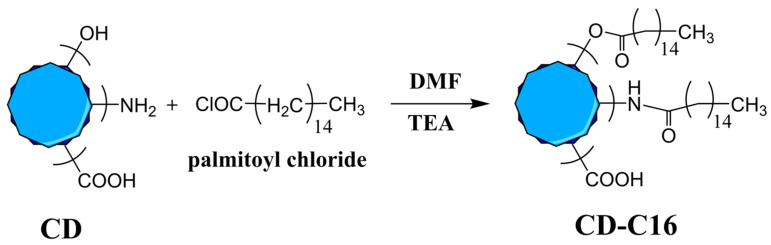
Schematic representation of nitrogen-doped carbon dots (CD) functionalization with alkyl chains following their interaction with palmitoyl chloride affording palmitoyl-functionalized carbon dots (CD-C16).

**Figure 1:**
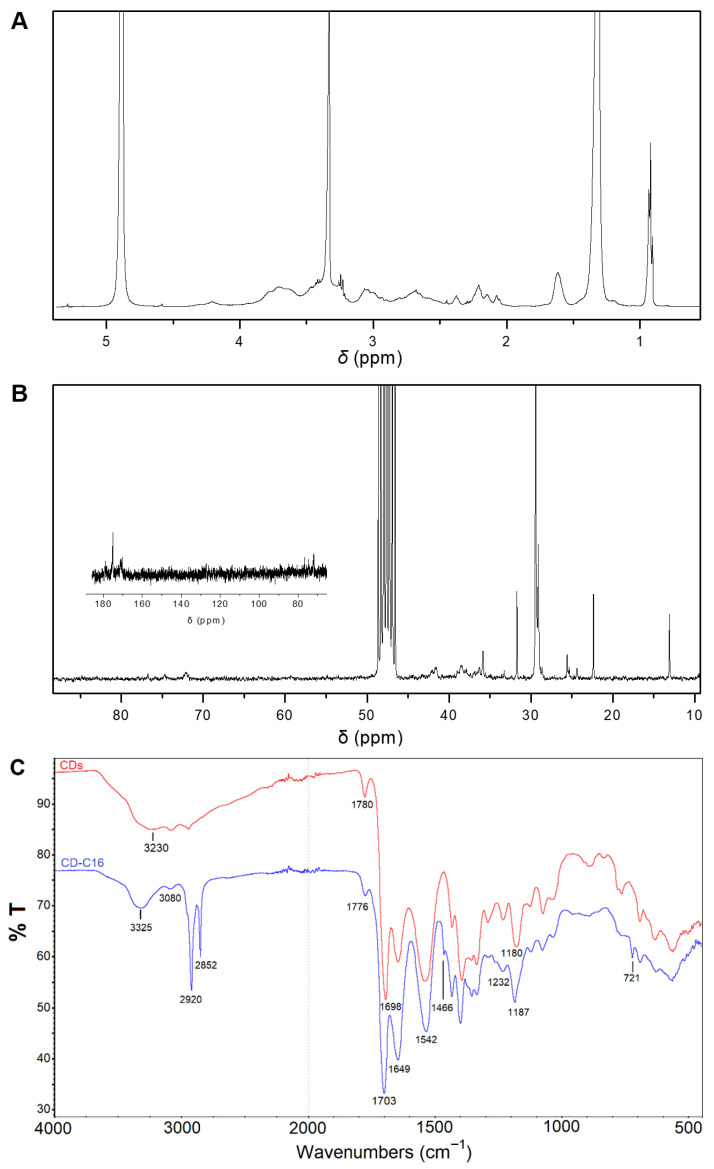
^1^H NMR (**A**) and ^13^C NMR (**B**) spectra of palmitoyl-functionalized carbon dots (CD-C16) as well as FTIR spectra (**C**) of parent nitrogen-doped carbon dots (CD) and of CD-C16 in the 4000–450 cm^−1^ region.

Functionalization of CD with palmitoyl (C16) alkyl chains was realized by the reaction in dry DMF of palmitoyl chloride with the amino and hydroxyl groups of CD in the presence of triethylamine (Scheme 1). After purification, the palmitoyl-functionalized carbon dots (CD-C16) were physicochemically characterized with ^1^H and ^13^C NMR, FTIR, UV-Vis, and fluorescence spectroscopies. The ^1^H NMR spectrum of CD-C16 (Figure 1A) provides clear evidence for the successful alkylation of CD with palmitoyl chains. Broad signals in the 2.0–2.3 ppm region as well as at 2.4 and 4.2 ppm are assigned to the protons of α-CH_2_ groups adjacent to the newly formed amide and ester groups (NHCOCH_2_, OCOCH_2_, and CH_2_OCO), respectively. Additional signals at 0.9, 1.3, and 1.6 ppm correspond to the protons of the terminal methyl groups, the methylene groups of the alkyl chains, and the β-CH_2_ groups, respectively. Signals observed in the 2.5–4.0 ppm region originate from CD and are attributed to protons attached to saturated carbon atoms adjacent to amino, amide, or oxygen-containing groups (Figure S1A, Supplementary Material) [43]. The successful alkylation of CD is further confirmed by ^13^C NMR spectroscopy (Figure 1B), as evidenced by the increased intensity of the signal at 175 ppm, which is attributed to the carbonyl carbons of the ester and amide groups, as well as by the appearance of the characteristic signals of the alkyl-chain carbons. Specifically, the peaks at 35.9, 29.1, and 25.6 ppm are assigned to the α-, β-, and γ-methylene carbons relative to the amide and ester bonds, respectively. Signals at 31.7 and 22.3 ppm are attributed to the β- and α-methylene carbons relative to the terminal methyl group, respectively, while the peak at 13.1 ppm corresponds to the terminal methyl carbon. In addition, the signal at 29.4 ppm is assigned to the remaining methylene carbons of the alkyl chains, clearly confirming the presence of palmitoyl chains in the final CD derivative. Moreover, the characteristic signals originating from CD are also observed, including peaks in the 35–45 ppm region attributed to aliphatic carbons and signals in the 170–180 ppm region corresponding to carbonyl carbons (C=O) (Figure S1B, Supplementary Material). Finaly, ^1^H-NMR spectroscopy was employed to estimate the palmitoyl content of CD using naphthalene as an internal standard (Figure S2, Supplementary Material). By comparing the integration of naphthalene aromatic protons at 7.5 and 7.9 ppm with that of the terminal methyl group protons of alkyl chains at 0.9 ppm, the alkyl chain content was found to be 1.93 mmol per gram of CD-C16, corresponding to ca. 50% *w*/*w* of alkylated groups in CD-C16. Complete functionalization of the primary amino groups of CD was further supported by the ninhydrin test, which is known to react with primary amino groups to produce an intense blue coloration. Application of the ninhydrin test to CD-C16 showed no color development, indicating the absence of unreacted amino groups. Since the determined palmitoyl content of CD exceeds the initial amino group content of CD, this result further indicates that palmitoyl chloride reacted not only with amino groups but also with hydroxyl groups of CD, which is consistent with the FTIR results (see below).

Infrared spectroscopy was also employed as an additional tool for the corroboration of the functionalization of CD with alkyl chains. As shown in Figure 1C, the FTIR spectrum of CD-C16 in the 4000–2500 cm^−1^ region, as compared to the respective spectrum of the parent CD, is characterized by the new strong peaks at 2920 and 2852 cm^−1^ of the antisymmetric and symmetric stretching modes of CH_2_ groups [48]. Of interest is the presence of the strong peak centered at 3325 cm^−1^, which is not prominent in the spectrum of unmodified CD, and is attributed to the NH stretching mode of the newly formed amide groups, at the expense of the peak at ~3230 cm^−1^ in the spectrum of CD, which is assigned to the NH stretching of its (unreacted) amino groups. Concurrently, the peak at 3080 cm^−1^, which is the overtone of the amide II band of the amide groups is, as expected, present in both parent CD and CD-C16. Overall, the broad infrared absorbance in the 3500–2500 cm^−1^ region of the CD spectrum, that can be tentatively assigned to the NH stretching of amino groups and the OH stretching vibrations of strongly hydrogen-bonded hydroxyl groups [48], is not evident in the spectrum of CD-C16, which is suggestive of their successful reaction with the alkyl chloride leading to amide and ester bonds formation, respectively. In the 1800–700 cm^−1^ region, the peak at 1780 cm^−1^ attributed to the C=O vibration of ester groups in the spectrum of CD shifts to 1776 cm^−1^ and becomes broader in the spectrum of CD-C16 as a result of new ester bonds formation. The peak at 1698 cm^−1^ attributed to the C=O vibration of the carboxylic groups in CD is shifted to the 1703 cm^−1^ due to the presence of newly formed hydrogen bonds of carbonyl groups with neighboring amide and ester groups. As expected, the amide I, II, and III bands are present in the spectra of both compounds at 1649, 1542 and 1232 cm^−1^, respectively. The bending as well as the rocking mode of the methylene groups of the alkyl chains at 1466 cm^−1^ and at 721 cm^−1^, respectively, are apparent as new peaks in the spectrum of CD-C16, confirming the presence of alkyl chains. Additionally, the band at 1180 cm^−1^ of C–O– stretching vibration of ester groups is shifted to the 1187 cm^−1^ and becomes sharper, further supporting the formation of new ester groups [48].

**Figure 2:**
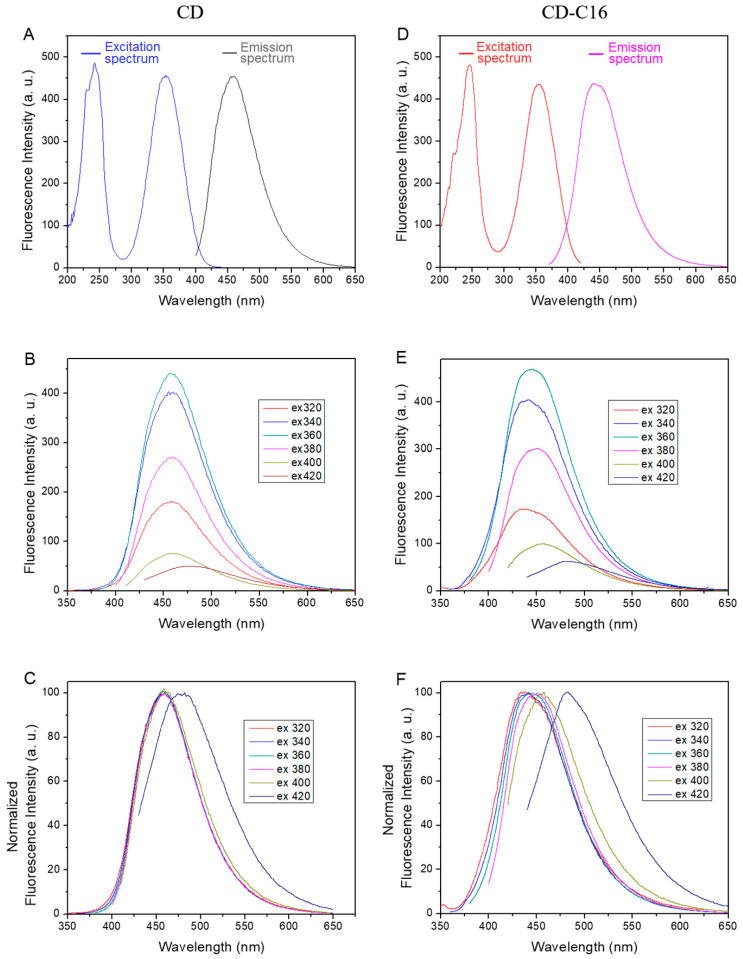
Fluorescence spectra of CD in water (**A**–**C**) and of CD-C16 in ethanol (**D**–**F**): excitation and emission spectra of CD (**A**) and of CD-C16 (the shoulders in the excitation spectra at 228 nm for CD or at 220 nm for CD-C16, are due to second-order diffraction of the light when the monochromator is set at 456 nm or 442 nm, respectively); emission spectra of CD (**B**) and of CD-C16 (**E**) at excitation wavelengths ranging from 320 nm to 420 nm (the intensities of the spectra after excitation at 420 nm are multiplied by 2 for better readability); the corresponding normalized emission spectra of CD (**C**) and of CD-C16 (**F**) at the various excitation wavelengths.

The fluorescence spectra of parent carbon dots, CD, are presented in Figure 2A–C, while the corresponding spectra of their alkylated derivatives, CD-C16, are shown in Figure 2D,F. It is evident that the alkylated carbon dots exhibit strong blue fluorescence after excitation at 350 nm, similar—but not identical—to that of parent CD. Their excitation spectrum in ethanol (Figure 2A) exhibits peaks at 246 nm and at 354 nm. Their position compared to those of parent CD in water that are registered at 242 nm and 352 nm, respectively, (Figure 2A) is almost the same, especially if we take into consideration the difference in the solvent employed. Therefore, we can also attribute the peak at 246 nm to the *n* → σ* (C-OH) or *π* → π* transitions of the carbon core, while the peak at 354 nm is attributed to the *π* → π* and *n* → π* (C=O and C=N) transitions of oxygen- and nitrogen-containing functional groups located at the surface of carbon cots [49,50]. The emission spectrum of CD-C16, however, is, in some way, different from the corresponding spectrum of CD: the band of the emission spectrum after excitation at 354 nm is observed at 442 nm (Figure 2B), in contrast to the corresponding band of parent CD registered at 456 nm. In addition, while monitoring the emission band after excitation at various wavelengths, (λ_ex_ from 320 to 420 nm) we observe (Figure 2B,C) that its maximum gradually shifts to higher wavelengths upon increasing λ_ex_ and, therefore, CD-C16 exhibit excitation-dependent fluorescence. On the contrary, it is known that citric acid-based CD typically display excitation independent behavior [49,50,51], and this is true also for the parent CD employed in this study (Figure 2D). These differences in the emission spectrum of CD-C16 compared to the spectrum of parent CD can be rationalized by taking into account that this band is considered to be the result of two overlapping peaks [50,51] and that the apparent excitation-independent emission of parent CD is the result of the properties of the band exhibiting the strongest emission. Indeed, the first one, with a fluorescence maximum at appr. 440 nm that does not depend on λ_ex_, was attributed to the presence of organic fluorophores, i.e., small molecular fluorescent species connected to the surface or incorporated inside the CD structure. The most prominent moiety is analogous to 1,2,3,5-tetrahydro-5-oxoimidazo[1,2-a]pyridine-7-carboxylic acid (IPCA), whose strong fluorescence does not depend on the excitation wavelength. In this connection, it should be noted that, by employing gel electrophoresis, it was demonstrated that the negatively charged fraction of carbon dots prepared from citric acid and ethylenediamine is bearing an IPCA-like fluorophore, which is responsible for the light emission that is both strong and excitation-independent [52]. On the other hand, the second one, having a maximum that continuously red-shifts upon increasing λ_ex_, was attributed to the emission of the carbogenic cores [51]. Evidently, the alkyl-chain functionalization of CD does not affect the fluorescence properties of groups located within the carbon core but moderates the fluorescence properties of surface organic fluorophores such as IPCA or ICPA-like moieties, possibly by the reaction of alkyl chloride with the secondary NH groups of the imidazole ring. This results in a fluorescence signal that is largely due to the carbogenic cores, indirectly confirming that primarily the small organic fluorophores of CD are functionalized under the conditions employed.

In agreement with the excitation spectrum of the carbon dots, the absorption spectrum of CD in water exhibits, as also designated in the literature, a main band centered at 350 nm attributed mainly to the *n* → π* transitions [49,50], and a shoulder at about 240 nm assigned to the *π* → π* transitions of the core carbon network [50,53]. The absorption spectrum of CD-C16 in ethanol shows that the main band is located at 352 nm, in line with the corresponding band of the parent carbon dots (for comparison both spectra are shown in Figure S3, Supplementary Material). On the other hand, the shoulder at 240 nm is hardly evident because of the newly formed –NH–(C=O)– groups, whose spectra are known to peak in this region and extend up to about 340 nm [54,55], thus resulting in the observed increased absorption of CD-C16 compared to CD in this region.

### 2.2. Preparation and Doxorubicin Encapsulation of Liposomal Systems Containing Hydrophilic (CD) or Lipophilic (CD-C16) Carbon Dots: Structural and Thermodynamic Properties

As widely applied in the literature, we prepared liposomal systems of ca. 100 nm, that are considered to enhance intratumoral liposomal drug accumulation due to the so-called enhanced permeation and retention (EPR) effect. According to this effect, liposomes of this size extravasate from the abnormal tumor microvasculature [10,56,57] and can easily diffuse throughout tumor tissue avoiding perivascular accumulation [10], thus achieving passive tumor targeting. For the development of thermosensitive liposomes, the dipalmitoylglycerophosphocholine (DPPC) phospholipid is typically employed due to its favorable main lipid phase transition temperature (~41 °C) [58], in tandem with the PEGylated phospholipid distearoylglycerophosphoethanolamine-polyethylene glycol (DSPE-PEG) at a 5% DSPE-PEG:DPPC molar ratio, that imparts long-circulation properties and stability in the biological milleu [30,57,59]. These lipids, in conjunction with the incorporation of either hydrophilic water-soluble carbon dots (CD) in their aqueous interior, or lipophilic carbon dots functionalized with alkyl chains (CD-C16) in their phospholipid bilayer, were used for the preparation of PEGylated DPPC liposomes of appr. 100 nm in diameter as detailed in the experiment section.

The effect of CD and CD-C16 on liposomes’ physicochemical properties, including hydrodynamic size and size distribution, z-potential, as well as phase transition characteristics, was investigated and followed by studies on their stability and bilayer permeability. Initial experiments on the effect of the newly synthesized lipophilic carbon dots (CD-C16) on the properties of PEGylated DPPC liposomes were performed to determine the maximum amount that could be incorporated into the bilayer while maintaining formulation stability. By employing various amounts of CD-C16 in the liposomal formulation (from 1% to 4% *w*/*w* with respect to total lipids) and monitoring their stability and bilayer permeability, we decided to use CD-C16 at a 2% *w*/*w* content. Amounts higher than 2.5% led to leaky formulations at 37 °C, as well as to very broad main lipid phase transitions, and therefore were excluded from any further studies. By quantification of the amount of CD-C16 in liposomes after the extrusion step, it was confirmed that the content of CD-C16 relative to total lipids was 2.1 ± 0.3% *w*/*w*, indicating that the lipophilic carbon dots were quantitatively embedded in the bilayer. Accordingly, we also prepared CD containing liposomes that also have a final CD content of 2% *w*/*w* with respect to total lipids, by selecting, after initial experiments, the appropriate concentration of CD in the hydration medium (see experimental section) that would lead to the same final CD content. Active loading of DOX in all the above liposomal formulations was attained using the standard pH gradient method [60,61,62] in order to evaluate the effect of hydrophilic or lipophilic carbon dots on bilayer properties, especially regarding their permeability at either 37 °C or at hyperthermia-related temperatures.

The presence of carbon dots either within the liposomal aqueous core or of the alkylated CD within the bilayer membrane necessitates the investigation of possible changes in their physicochemical properties including their effect on z-potential and size distribution as compared to the parent liposomes. The surface charge of parent liposomes in RPMI was found to be −12.6 ± 0.5 mV, as expected, due to the presence of the negatively charged DSPE-PEG in the bilayer, which endows liposomal stability through both electrostatic repulsion and steric stabilization from PEG chains. After DOX loading and the respective purification step, the registered z-potential is practically unaffected (−12.3 ± 0.6 mV). The presence of carbon dots in their aqueous interior or alkylated carbon dots in the bilayer also results in similar values, i.e., −12.3 ± 1.3 mV for empty CD-C16 containing liposomes in RPMI, and −13.4 ± 0.9 mV for the corresponding DOX-loaded liposomes. Overall, the variation in electrophoretic mobility of all liposomal systems is minimal. This can be justified by the fact that the employed CD exhibit a slightly positive value (2.5 ± 1.2 mV) [40,43], while their CD-C16 derivative (although not water soluble, which precludes their electrophoretic mobility measurements in water) is not anticipated to have a significant surface charge.

**Figure 3:**
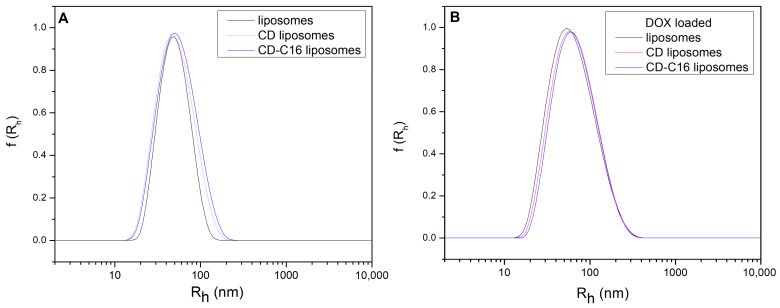
Intensity-weighted hydrodynamic radii distributions at room temperature of PEGylated dipalmitoylglycerophosphocholine (DPPC) liposomes, as well as CD and CD-C16 containing liposomes, either empty (**A**) or doxorubicin (DOX)-loaded (**B**). Data shown are the mean of at least three independent dynamic light scattering measurements.

Intensity-weighted hydrodynamic radii of non-loaded PEGylated DPPC liposomes, as well as CD and CD-C16 containing liposomes, determined by employing dynamic light scattering (DLS) experiments (Figure 3A), reveal a rather narrow distribution centered in all cases at 48 ± 2 nm; CD-C16 containing liposomes are marginally larger (51 ± 2 nm), which could be the result of either a more flexible bilayer membrane, or of the need for larger curvature of the bilayer due to the presence within the bilayer of the bulky alkylated carbon dots. Size distributions of DOX-loaded liposomes, after removal of the non-encapsulated DOX employing a Sephadex column, were found to be somehow broader and centered at 56 ± 3 nm (Figure 3B); DOX-loaded CD and CD-C16 containing liposomes also reveal a similar trend of having a slightly larger radii (i.e., ca. 58 ± 3 nm), which, however, is not statistically significant.

**Figure 4:**
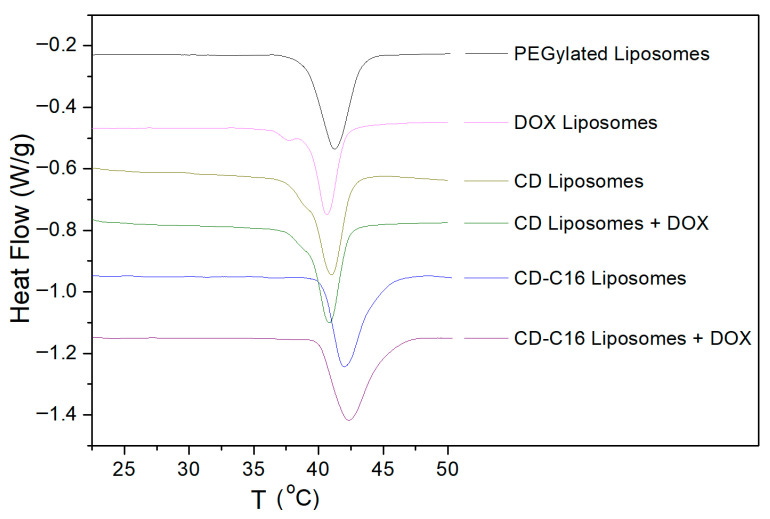
Total heat flow DSC profiles of PEGylated DPPC liposomes, as well as CD and CD-C16 containing liposomes, either empty or loaded with DOX, in RPMI during the first heating cycle (endo down). The heating scan rate for all of thermographs was 1 °C/min. The thermograms are shifted vertically for clarity.

Differential scanning calorimetry (DSC) experiments were used to detect changes in the thermodynamic properties of the lipid main phase transition and the possible interaction of carbon dots with the phospholipid bilayer structure. As shown in Table 1 and Figure 4, the parent liposomes have a *T*_m_ at ca. 41°C in line with the literature values [58,63,64]. The presence of hydrophilic carbon dots in the aqueous interior does not affect the main lipid phase transition temperature, given that the *T*_m_ values are, within experimental error, essentially the same during both the first and second heating run. The Δ*T*_½_ value (i.e., the width at half maximum of the DSC peak) that is designating the cooperative melting of the hydrocarbon chains of the lipid molecules, is slightly lower in the presence of CD, suggesting a small increase in cooperativity. This is pointing to some favorable interaction of the phospholipid polar groups with hydrophilic CD that result in an increase in the number of lipids in each cooperative unit during melting [65].

On the other hand, the presence of lipophilic carbon dots in the bilayer imparts a pronounced effect: both the *T*_m_ and the width at half maximum of the DSC peak increase substantially. Drawing conclusions from the effect of proteins on phospholipid bilayers, in particular of proteins embedded within the membrane, we tentatively attribute the observed broadening of main lipid phase transition to the mixing of carbon dots alkyl chains with the lipophilic part of the phospholipids that are located near the alkylated carbon dots. The observed increase in transition temperature, together with the broadening of the heat capacity profiles, is reported in cases where proteins are dissolved into the gel phase (due to favorable interactions) but not in the fluid phase where, due to unfavorable interactions, the proteins instead form aggregates [65]. In an analogy, we propose that upon melting of the lipid chains of the bilayer towards the liquid crystalline phase, the embedded lipophilic carbon dots are not any more solubilized to the same extend as in the gel phase, forming carbon-dots rich subphases, which could provide a justification for the observed permeability increase (as shown below in Section 2.3).

### 2.3. The Effect of Hydrophilic (CD) or Lipophilic (CD-C16) Carbon Dots on Time- and Temperature-Dependent Drug Release of Liposomal Systems

**Figure 5:**
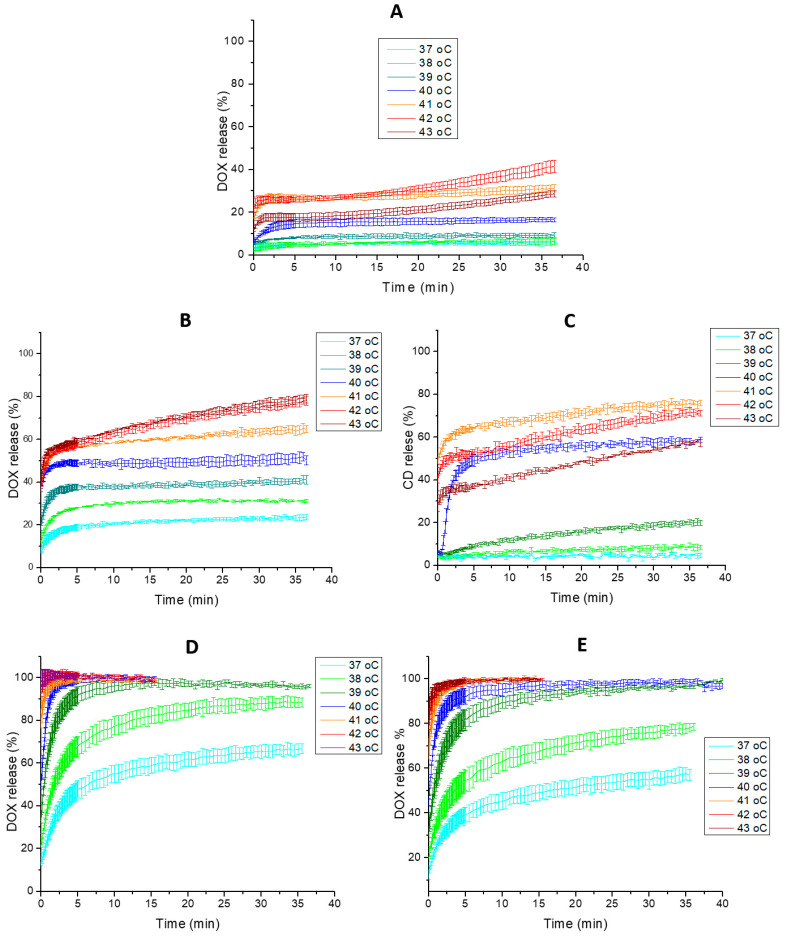
Time- and temperature-dependent release from drug-loaded liposomal formulations at various temperatures, from 37 to 43 °C. (**A**) DOX release from parent non-thermal sensitive PEGylated DPPC liposomes in RPMI; (**B**) DOX release from CD containing liposomes in RPMI; (**C**) CD release from CD containing liposomes in DPBS; DOX release from CD-C16 containing liposomes in RPMI (**D**) or in DPBS (**E**). Measurements were taken every 0.2 s during the first five minutes of the incubation period, and every 1 min afterwards. All data points are the mean values of at least two independent experiments.

The developed series of DOX-loaded liposomal formulations were evaluated with regard to stability and drug release properties at physiological temperatures (37 °C) and at elevated temperatures (38–43 °C) in RPMI, in order to have exactly the same aqueous medium as in the subsequent cell culture viability experiments (Section 3.7). Continuous monitoring of drug release over time is feasible because DOX is strongly fluorescent at low concentrations (i.e., upon its release from the liposomal interior in the external aqueous phase), whereas its fluorescence signal is strongly quenched at high concentrations (i.e., at concentrations achieved in the liposomal interior by employing active drug loading). This property is not uncommon for fluorescent molecules, and indeed this is also the case for carbon dots. Thus, it was possible to monitor, in addition, the release of encapsulated CD from the respective liposomes at various temperatures versus time. However, in this case, their release was monitored in Dulbecco’s modified Phosphate-Buffered Saline, suitable for cell culture (DPBS). This was necessary, as at the CD excitation wavelength of carbon dots (350 nm), RMPI absorbs strongly, in contrast to DPBS which is transparent in this region. In order to detect if there is any significant alteration in DOX release properties between the two different culture media, DOX release from CD-C16 containing liposomes dispersed in both media was also determined (Figure 5).

DOX release from parent liposomes is, as expected, very limited even at 41 °C (less than 30%, Figure 5A) since there are no additional molecules present in the bilayer that could induce membrane fluidity or pore opening near *T*_m_. The stability of the bilayer is primarily manifested by the extremely low DOX release at 37 °C (ca. 5%). At temperatures above 42 °C after ca. 20 min, the destabilization of the bilayer we observed manifested as a gradual increase in release rate. This may be attributed to interactions of membrane lipids with components of the RPMI medium, given that such behavior was not reported for this system in release experiments performed in water or in simple phosphate-buffered saline [62,64].

CD containing liposomes were found to be rather stable at 37 °C, as DOX release attains a plateau value of ~20% after 20 min (Figure 5B). Upon temperature increase we observe DOX release reaching plateau values within the first 5 min. At 40 and 41 °C, a 50 to 60% DOX release is evident, while at 42–43 °C a continuous increase in DOX release is observed, probably due to the interactions of lipids with RPMI components as observed above for the parent liposomes. It is of interest to note that at 40 °C, the temperature employed in the following cell in vitro experiments (see Section 2.4), 50% of DOX is released within the first 5 min, remaining constant until the end of the experiment.

For this system, we also utilized the property of CD as a self-quenching material to detect its release from CD containing liposomes, as well as from the DOX-loaded counterparts, following the same as above procedure. As mentioned above, CD release experiments in RPMI were not possible, and therefore the release of the encapsulated carbon dots as a function of time and temperature was performed in DPBS (Figure 5C). While negligent release was observed at 37 and 38 °C, at elevated temperatures CD release gradually increases. At 40 °C, about 50% of encapsulated CD is released in the medium which could rationalize why the liposomes are no longer thermosensitive, impeding the complete release of DOX at this temperature.

On the other hand, in CD-C16 containing liposomes at 39–40 °C, DOX is released by more than 95% within the first 5 min either in RPMI or in DPBS (Figure 5D,E). At even higher temperatures (41–42 °C) a much faster and complete release within the first 1–2 min is observed. This suggests that the bilayer is becoming “leaky” at temperatures lower than the registered *T*_m_ (42 °C). This is in line with the rather broad width of the lipid main phase transition registered in DSC experiments (cf. Figure 4), providing further proof of a strong perturbation of the membrane by the presence of lipophilic carbon dots. This membrane destabilization is also evident in the observed release profile at 37 °C, where up to 60% DOX is released within 35 min.

### 2.4. In Vitro Cell Viability Studies of DOX-Loaded Liposomal Formulations Against a Cancerous and a Non-Cancerous Cell Line

For the initial assessment of the efficacy of the developed liposomal formulations as anti-neoplastic thermosensitive drug delivery systems, we employed a DOX sensitive non-cancerous cell line, HEK293 [41], and a cancerous cell line, PC3, which is known to be DOX resistant [42,66]. The effect on cell viability of DOX, either free or encapsulated in the thermosensitive CD or CD-C16 containing liposomes, was tested at either 37 or 40 °C employing DOX at 5 and 10 μΜ for the PC3 cells, and at 1 μΜ for the HEK293 cells. For comparison purposes, in addition to the controls (untreated cells), cells were also treated with non-drug-loaded parent liposomes, as well as with CD and CD-C16 containing liposomes serving as additional controls, at the same concentrations as those employed in liposomal DOX-loaded formulations (corresponding to the liposomal concentration in experiments at the highest DOX concentration). The incubation time was chosen to be 1 h given that this time frame is typically employed in clinical applications of hyperthermia (about 1 h at the treated site) [4,5,67,68]. Following this brief incubation time, drug-containing media were removed from all well-plates, replaced with complete media, and cells were allowed for incubation at 37 °C for 24 h, before assessment of cell viability employing the MTT assay.

**Figure 6:**
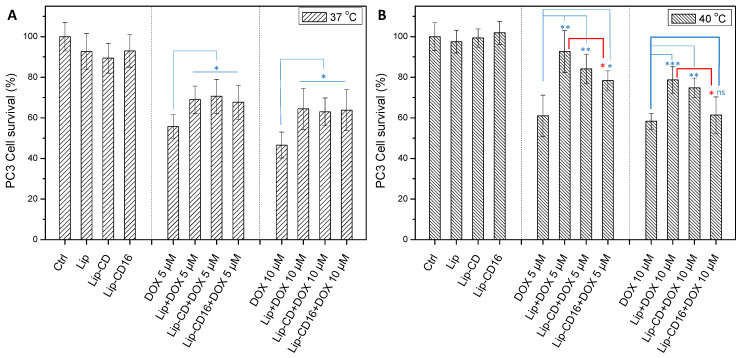
Comparative cytotoxicity of either empty or DOX-loaded PEGylated DPPC liposomes (Lip), or CD (Lip-CD) and CD-C16 (Lip-CD16) containing liposomes against PC3 cells following an 1 h incubation at (**A**) 37 or (**B**) 40°C. DOX concentration was 5 or 10 μM, while the concentration of empty liposomes corresponded to the liposomal concentration in experiments at the highest DOX concentration (10 μΜ). The cytotoxicity was assessed after a 24 h post-incubation period employing the standard MTT assay. Cell survival data are the mean of at least six independent values while error bars represent one standard deviation. The statistical significance was derived by Student’s *t*-test following the assignment: * *p* < 0.05, ** *p* < 0.01, *** *p* < 0.001; ns denotes no statistical significance, *p* > 0.05.

Against PC3 cells, all empty liposomal formulations have a non-statistically significant cytotoxic effect compared to control, while free DOX at 5 and 10 μM after incubation either at 37 or 40 °C exhibits a 40 or 60% cell viability, respectively (Figure 6). DOX-loaded conventional liposomal formulations at 37 °C (Figure 6A) are less cytotoxic compared to free DOX (*p* < 0.05), which is to be expected since it is well known that liposomal DOX is less potent compared to free DOX, as discussed in more detail in the Introduction section, due to the limited release of entrapped DOX. In this sense, all DOX-loaded liposomal formulations (either thermosensitive or not) have the same moderate cytotoxic effect on PC3 cells, depending slightly on the concentration of encapsulated DOX (cell viability of ca. 70% at 5 μM, vs. ca. 60% at 10 μM of DOX). On the other hand, at 40 °C the thermosensitivity of CD and CD-C16 containing liposomes on cell viability comes into effect: at 5 μΜ DOX concentration, drug-loaded CD and CD-C16 containing liposomes are more potent compared to the parent DOX-loaded liposomes (*p* < 0.05), though still less potent than free DOX (i.e., cell viability of CD-C16 containing liposomes is 75% compared to 60% for DOX, *p* < 0.05). However, at 10 μΜ DOX concentration, both thermosentive formulations are more toxic compared to non-thermosensitive liposomes (*p* < 0.05), while CD-C16 containing liposomes are equally potent to free DOX (Figure 6B). These results clearly indicate that incorporation of CD-C16 within the liposomal bilayer—while not inherently cytotoxic by itself—enhances the cytotoxic efficacy of encapsulated DOX under hyperthermia conditions (40 °C), but not at the physiological temperature. At this temperature (37 °C), its activity remains comparable to that of conventional, non-thermosensitive, liposomal formulations and significantly lower than that of free DOX.

**Figure 7:**
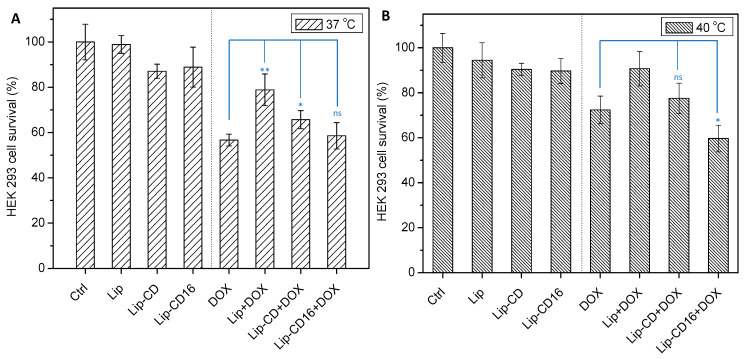
Comparative cytotoxicity of either empty or DOX-loaded PEGylated DPPC liposomes (Lip), or CD (Lip-CD) and CD-C16 (Lip-CD16) containing liposomes against HEK293 cells following an 1 h incubation at (**A**) 37 or (**B**) 40°C. DOX concentration was 1 μM, while the concentration of empty liposomes corresponded to the liposomal concentration in experiments at this DOX concentration (1 μΜ). The cytotoxicity was assessed after a 24 h post-incubation period employing the standard MTT assay. Cell survival data are the mean of at least six independent values, while error bars represent one standard deviation. The statistical significance was derived by Student’s *t*-test following the assignment: * *p* < 0.05, ** *p* < 0.01; ns denotes no statistical significance, *p* > 0.05.

In order to have a preliminary indication on whether there is a difference in the cytotoxicity of these systems against DOX-resistant and DOX-sensitive cells, we also tested these formulations against HEK293 cells, a DOX sensitive non-cancerous cell line. It was of interest to find out that DOX-loaded liposomal formulations are potent both at 37 and 40 °C after treating the cells for 1 h at a DOX concentration of 1 μM (Figure 7). Specifically, at 37 °C all DOX-loaded liposomal formulations exert a noteworthy cytotoxic effect on HEK293 cells. In this case, the thermosensitive liposomes were more potent than conventional liposomes—in contrast to PC3 cells where all formulations were equally potent (Figure 7A). In particular CD-C16 containing liposomes exert the same cytotoxic effect as free DOX (ca. 55%), although only 50% of DOX is released at this temperature as shown by the corresponding release profile. Therefore, we can tentatively assume that this is an indication that these formulations are more easily internalized within these cells, which possibly explains their significant DOX activity. At 40 °C (Figure 7B), the thermosensitive formulations are even more cytotoxic: CD containing liposomes are equally toxic to free DOX (ca. 75%), while CD-C16 containing liposomes are even more potent than free DOX (60 vs. 72%, *p* < 0.05), attributed to both the thermosensitivity of these liposomal systems and the enhanced internalization, especially in the case of the CD-C16 system. This significant activity of CD-C16 containing liposomes is, however, counterweighed by the considerable cytotoxicity of this system at 37 °C. Consequently, in this case, the use of CD containing liposomes could be preferable to CD-C16 containing liposomes in hyperthermia therapeutic schemes, since at 40 °C the former are equally potent to free DOX and less toxic at 37 °C. More importantly this points to the need for additional experiments and extensive research on the effect of these thermosensitive systems against DOX sensitive cells.

## 3. Materials and Methods

### 3.1. Chemicals and Reagents

Citric acid (99.8%), ethylenediamine (≥99%), palmitoyl chloride (98%), and triethylamine (TEA, ≥99%) were purchased from Sigma-Aldrich Ltd. (Poole, UK). The phospholipids 1,2-dipalmitoyl-sn-glycero-3-phosphocholine (DPPC) were obtained from Lipoid GmbH (Ludwigshafen, Germany), while 1,2-distearoyl-sn-glycero-3-phosphoethanolamine-*N*-[methoxy(polyethylene glycol)-2000] ammonium salt (DSPE-PEG) was obtained from Avanti Polar Lipids (Alabaster, AL, USA). Doxorubicin hydrochloride (DOX, >98%) was purchased from Thermo Fisher Scientific (Waltham, MA, USA). Nucleopore filters of 100 nm pore size (Whatman, Maidstone, UK) were employed for liposome extrusion. Sephadex G-25 (fine) was obtained Sigma–Aldrich (St. Louis, MA, USA). Dulbecco’s Phosphate-Buffered Saline, which was modified to be suitable for cell culture (DPBS) and RPMI 1640 without L-glutamine and without phenol red (RPMI) that were employed for the DOX release experiments, was purchased from Biowest (Nuaillé, France).

For the biological evaluation experiments, RPMI 1640 with stable L-glutamine and with 25 mM HEPES, fetal bovine serum (FBS), penicillin (100 U/mL)/streptomycin (100 μg/mL) solution, trypsin (0.05% *w*/*v*)/EDTA (0.25% *w*/*v*) solution, and Dulbecco’s Phosphate Buffered Saline without Calcium and Magnesium were purchased from Biowest (Nuaillé, France). Thiazolyl blue tetrazolium bromide (MTT) was purchased from Sigma-Aldrich Ltd. (Poole, UK). High-purity dimethyl sulfoxide (DMSO) was obtained from Merck KGaA (Calbiochem^®^, Darmstadt, Germany). All other reagents and solvents were of analytical grade and were used without further purification.

### 3.2. Synthesis and Characterization of Alkylated Nitrogen-Doped Carbon Dots

The preparation of highly luminescent nitrogen-doped CD by microwave irradiation from an aqueous solution of citric acid and ethylenediamine (molar ratio of 0.90:1) was based on previously published protocols [69], and is described in detail in our previous publication [43]. The presence of carboxylic, hydroxyl, and amino groups on the surface of the resulting CD nanoparticles was established by ^1^H NMR and ^13^C NMR spectroscopy (Figure S1A,B) employing a Bruker Avance DRX spectrometer operating at 500 and 125.1 MHz, respectively (Bruker Biospin GmbH, Rheinstetten, Germany). Taking advantage of the presence of hydroxyl and primary amino surface groups of CD, their functionalization with alkyl chains was realized by the step-wise addition of palmitoyl chloride (1.2 mmol) in a dry DMF solution of CD (100 mg) at 0°C in the presence of 0.5 mL TEA under rigorous stirring and argon atmosphere. The reaction mixture was allowed to reach room temperature and further heated at 40 °C for 24 h. After precipitation with diethyl ether to remove unreacted palmitoyl chloride, the crude product was dried and further treated with water to eliminate any unreacted CD. The obtained dry material was characterized by ^1^H NMR and ^13^C NMR spectroscopy (Figure 1A,B), while the absence of primary amino groups in CD-C16 was corroborated by employing the ninhydrin test. Furthermore, by employing naphthalene as the internal standard and comparing the integrations of naphthalene aromatic protons with that of the terminal methyl group protons of the alkyl chains, it was found that 1.93 mmol of C16 alkyl groups was grafted per 1 g of CD (Figure S2).

^1^H-NMR (500 MHz, CD_3_OD), *δ* (ppm): 4.2 (CH_2_OCO), 4.0–2.5 (CH_2_ of DC), 2.4 (OCOCH_2_), 2.0–2.3 (NHCOCH_2_), 1.6 (NHCOCH_2_CH_2_, OCOCH_2_CH_2_), 1.3 (CH_2_ of palmitoyl chain), 0.9 (CH_3_).

^13^C-NMR (125.1 MHz, CD_3_OD), *δ* (ppm): 180–170 (CO of CD), 175 (OCO, NHCO), 76.7, 74.7 and 72.5 (CH_2_O), 36–43 (CH_2_ of CD), 35.9 (OCOCH_2_, NHCOCH_2_), 31.7 (CH_2_CH_2_CH_3_), 29.1 (OCOCH_2_CH_2_, NHCOCH_2_CH_2_), 25.6 (OCOCH_2_CH_2_CH_2_, NHCOCH_2_CH_2_CH_2_), 22.3 (CH_2_CH_3_), 13.1 (CH_3_).

FTIR spectra were obtained by averaging 64 individual scans at a resolution of 4 cm^−1^, employing a Nicolet 6700 spectrometer (Thermo Scientific, Waltham, MA, USA) with a Specac Quest diamond ATR accessory (Specac Ltd., Orpington, Kent, UK). The excitation and emission spectra of CD-C16 in ethanol were recorded employing a Cary Eclipse fluorescence spectrophotometer (Varian Inc., Mulgrave, Victoria, Australia) and were compared to the corresponding spectra of parent CD obtained in water. UV-Vis spectra were recorded using a Cary 100 Conc spectrophotometer (Varian Inc., Mulgrave, VIC, Australia).

### 3.3. Preparation of Liposomes, CD Containing Liposomes, or Alkylated CD-C16 Containing Liposomes, and Doxorubicin Active Loading Therein

Unilamellar DPPC:DPSE-PEG liposomes of 100 nm diameter were prepared by the extrusion method employing a LiposoFast-Pneumatic laboratory extruder (Avestin Inc., Ottawa, ON, Canada) [70]. In a typical experiment, 20.0 mg of DPPC and 3.82 mg of DSPE-PEG (5% molar with respect to DPPC) were dissolved in a 2:1 *v*/*v* chloroform/methanol solution. Alternatively, for the preparation of the alkylated CD-C16 containing liposomes 2% *w*/*w* of CD-C16 with respect to total lipids, the corresponding amount of CD-C16 was dissolved in the chloroform/methanol solution. The solvents were evaporated under reduced pressure in a rotavapor at 30 °C and the lipid film formed was kept under high vacuum for 18 h to remove any residual solvent. The lipid film was then hydrated with citrate buffer (2 mL, 150 mM, pH = 4.2) at 50–55°C for 30 min under argon atmosphere or with a solution of CD in the citrate buffer (10 mg/mL) for the preparation of CD containing liposomes. The obtained suspension was thermostated at 50 °C and was extruded, applying 25 cycles, through two stacked polycarbonate filters of 100 nm pore size.

DOX was loaded according to the pH-gradient active-loading protocol [60,61,62]. Specifically, after the extrusion step, the aqueous citrate solution of the liposomal dispersions was exchanged with DPBS, employing a Sephadex G-25 size exclusion minicolumn preconditioned with DPBS, following the protocol detailed in reference [31]. After establishing the pH gradient, 1 mg of DOX was added to 2 mL of liposomal dispersions and the dispersion was allowed at 37 °C for 30 min under argon atmosphere. After cooling to room temperature, liposomes were again passed through a Sephadex G-25 minicolumn as above, conditioned with either RPMI or DPBS, to remove non-encapsulated DOX and afford DOX-loaded liposomes dispersed in cell culture medium. Finally, empty (non-DOX loaded) liposomes were also prepared to be used as control. Encapsulated DOX concentration was determined employing UV-Vis spectroscopy. To this end, 50 μL of liposomal dispersions were added to 3 mL of DPBS and, after the addition of 10 μL of Triton X-100 (10% *w*/*w*) solution to obtain a clear solution and complete release of DOX, its absorption was registered at 480 nm.

UV-Vis spectroscopy was employed for the quantification of CD encapsulated in the final CD containing liposomes: in this case, 100 μL of liposomal dispersions were added to 3 mL of DPBS and the absorption of the resulting clear solution after the addition of 10 μL of Triton X-100 (10% *w*/*w*) solution was registered at 352 nm. The final DOX and CD concentrations were derived from the standard calibration curves that were independently generated for DOX and CD in DPBS. DOX concentrations in all preparations were found to be 400 ± 50 μg/mL and CD concentrations were 230 ± 30 μg/mL. The total lipid weight in liposomes was 11.0 ± 0.4 mg/mL, while the calculated final CD/total lipid ratio was 2.0 ± 0.2% *w*/*w*. For the quantification of CD-C16 in the respective liposomal formulations, 100 μL of liposomes was added to 1 mL of ethanol:water 90:10 *v*/*v*, and the solution absorbance at 354 nm was registered. CD-C16 concentration was determined by employing a standard calibration curve of CD-C16, also in the same solvent system. It was found that CD-C16 concentration was 0.25 ± 0.04 mg/mL, while the total lipid weight in the respective formulations was 11.8 ± 0.5 mg/mL, leading to a CD-C16/total lipid ratio of 2.1 ± 0.3% *w*/*w*.

### 3.4. Characterization Techniques

All prepared liposomal formulations were stored at 4 °C and investigated within the next 24 h employing z-potential, dynamic light scattering (DLS), and differential scanning calorimetry (DSC). The z-potential values were obtained at 23 °C using a ZetaPlus instrument (Brookhaven Instruments Corp., Long Island, NY, USA) equipped with a 35 mW solid-state laser emitting at 660 nm. From the obtained electrophoretic mobility, the z-potential values were obtained using Smoluchowski’s equation. Typically, 100 μL of liposomes were diluted to 1.8 mL with RPMI and introduced into the instrument cell. Ten measurements were collected for each dispersion and the results were averaged. The mean hydrodynamic radii and size distribution of liposomes were determined at 23 °C employing a dynamic light scattering apparatus (AXIOS-150/EX, Triton Hellas, Thessaloniki, Greece) equipped with a 30 mW laser source at 658 nm and an Avalanche photodiode detector at an angle of 90° (data acquisition time 20 s). For these experiments, 50 μL of liposomes were diluted with 200 μL of RPMI and 10 measurements were acquired for each dispersion. To obtain the apparent hydrodynamic radii distribution, the respective autocorrelation functions were analyzed using the CONTIN software package [71] provided by the manufacturer of the DLS apparatus.

The thermodynamic parameters related to the main lipid phase transition including the transition temperature, *T*_m_, and the temperature width at half maximum of the DSC peak, Δ*T*_½_, were determined by employing a MDSC 2920 calorimeter (TA Instruments, New Castle, DE, USA) under nitrogen flow (20 mL/min), using a heating rate of 1 °C/min and a temperature modulation amplitude of 0.15 °C every 60 s. For the determination of *T*_m_ values, the embedded TA Instruments Universal Analysis 2000 software (version 3.0G) was employed, while for the determination of Δ*T*_½_ the OriginPro^®^ software (version 8, Microcal Inc., Northampton, MA, USA) was used. Liposomal dispersions (100 μL) were centrifuged at 40,000 *g* for 60 min at 20 °C in a Kubota 7780 centrifuge (Kubota Corporation, Tokyo, Japan) and the resulting wet pellet was collected, transferred, and hermetically sealed into aluminum DSC pans [26]. Two heating/cooling scans were performed between 15 °C and 55 °C for each formulation. Data presented are the mean values of two independent liposomal formulations. The deviation of the *T*_m_ and Δ*T*_½_, values were ±0.2 °C.

### 3.5. Bilayer Membranes Permeability: Temperature and Time-Depended Release of Encapsulated DOX and CD

For the determination of DOX permeability through the liposomal membranes versus time, we follow the procedure detailed in [31], which takes advantage of the significantly reduced DOX fluorescence when encapsulated within the liposomal interior due to its self-quenching properties at high concentrations. Accordingly, the initial fluorescence signal of DOX-loaded liposomes, after the successful removal of non-encapsulated DOX, is extremely low; upon DOX release in the aqueous medium an increase in the fluorescence intensity is observed as DOX diluted in the outer bulk medium is no more self-quenched. Additionally, by choosing the correct volume of liposomal dispersion added in the fluorescence cell we ensure that the maximum concentration of DOX at 100% release in the cell is within the linear part of the calibration curve of DOX. Therefore, the concentration of DOX in the outer medium versus time at various temperatures was monitored by registering the DOX fluorescence intensity of liposome dispersions using a Cary Eclipse spectrophotometer (Varian Inc., Mulgrave, VIC, Australia) equipped with a Cary Single Cell Peltier accessory (type SPVF-1 × 0, Varian Inc., Mulgrave, VIC, Australia) able to control and stabilize the temperature in the cell within 0.1 °C under continuous stirring. For each experiment in a previously thermally equilibrated cell at the predetermined temperature containing 2.7 mL of DPBS or RPMI DOX-loaded liposomes (10 μL) were added, and fluorescence intensity measurements were initiated. The initial fluorescence intensity I_0_ of each sample was determined at 25 °C in a separate experiment employing the same as above experimental conditions. The fluorescence intensity over time (I_t_) at 592 nm (λ_ex_ = 492 nm) was monitored at specific time intervals for a total of 40 min. No photobleaching took place throughout the experiment as the xenon flash lamp is only active when a data point is acquired. At the end of the experiment, 10 μL of 10% Triton X-100 was added in order to solubilize liposomes and drive all DOX in the aqueous media to acquire I_max_, which was considered as 100% release. DOX release vs. time was calculated as Release (%) = (I_t_ − I_0_)/(I_max_ − I_0_) × 100. Corrections were made for the effects of temperature on DOX fluorescence intensity.

We utilized the property of CD as a self-quenching material to detect its release from CD-loaded and CD-and-DOX-loaded liposomes, following the same as above procedure. For these experiments, 20 μL of liposomes encapsulating carbon dots in their interior were added to 2.7 mL of DPBS and fluorescence intensity measurements over time were registered at 456 nm after excitation at 362 nm, following the procedure as described above for DOX release experiments. It should be noted that CD release experiments in RPMI were not possible, as RPMI medium strongly absorbs at the excitation wavelength of CD, thereby prohibiting fluorescence measurements.

### 3.6. Cell Culture and Treatments

The human prostate cancer PC3 cell line as well as the non-cancerous human embryonic kidney HEK293 of the cell bank from the Institute of Nanoscience and Nanotechnology, NCSR Demokritos, were purchased from the American Type Culture Collection (ATCC CRL-1435™ and CRL-1573™, respectively; Manassas, VA, USA). Cells were grown in an RPMI 1640 medium supplemented with 10% FBS and 1% penicillin/streptomycin at 37 °C in a 5% CO_2_ humidified atmosphere and sub-cultured, twice a week, after detaching with a trypsin solution. The cells were free of mycoplasma contamination, as ascertained by regular fluorochrome-staining microscopy tests using 4′,6′-diamidino-2-phenylindole (DAPI, Sigma-Aldrich).

### 3.7. Cell Viability Assay

The cell viability of empty and DOX-loaded liposomal formulations against PC3 and HEK293 cell lines was assessed employing the MTT assay. PC3 cells were seeded in 96-well plates (3 × 10^3^ cells per well in 100 μL culture medium) and incubated at 37 °C in a 5% CO_2_ atmosphere. After 24 h, cells were treated with free DOX and DOX-loaded liposomes at two different DOX concentrations (5 mM and 10 μΜ) and incubated at either 37 °C or 40 °C for 1 h in an RPMI medium free of FBS. HEK293 cells were treated as described above at DOX concentrations of 1 μΜ due to their high sensitivity to DOX. In addition to the control (untreated cells), for comparison purposes, cells were also treated with empty PEGylated DPPC liposomes, as well as with empty CD and CD-C16 containing liposomes at liposomal concentrations equivalent to those used in experiments with the DOX-loaded liposomes at 10 μΜ DOX for PC3 cells or at 1 μM DOX for the HEK293 cells. After the one-hour incubation period, the cells were washed with PBS, and RPMI 1640 medium supplemented with 10% FBS and 1% penicillin/streptomycin (complete medium) was added in each well and further incubated for the next 24 h at 37 °C in a 5% CO_2_ humidified atmosphere. Afterwards, cell media were replaced with complete medium containing MTT (1 mg/mL) and incubated at 37 °C in a 5% CO_2_ humidified atmosphere for 4 h. The resulting formazan crystals were solubilized with DMSO (100 μL per well, shaking for 5 min at 100 rpm in an orbital shaker). The absorbance was measured with an Infinite M200 plate reader (Tecan Group Ltd., Mannedorf, Switzerland) at a wavelength of 540 nm. Background absorption was measured at 620 nm and subtracted. The mitochondrial redox function (translated as cell viability) was calculated as the survival percentage compared to cells that were treated only with complete medium (control). Blank values measured in wells with DMSO and no cells were in all cases subtracted. Six replicates were performed for each concentration, and the experiments were repeated in triplicate. The data are presented as mean ± standard deviation. A Student’s *t*-test was employed to assess the statistical significance for all treatments (* *p* < 0.05, ** *p* < 0.01, *** *p* < 0.001, ns: *p* > 0.05).

## 4. Conclusions

In our previous study, it was shown that carbon dots interact with the phospholipid membrane of liposomes, affecting drug membrane permeability, and also that their presence in the culture medium enhance free drug internalization in cancer cells, suggesting that they also affect cell membrane permeability. In this work, the interaction of carbon dots with biological membranes was utilized for the development of thermosensitive liposomal drug delivery systems, encapsulating the well-known anticancer drug doxorubicin (DOX). Specifically, the incorporation of hydrophilic carbon dots within the liposomal aqueous core, or of alkylated lipophilic carbon dots within the bilayer membrane, affords systems with drug release properties at 40 °C, i.e., at mild hyperthermia relevant temperature. DLS, DSC, and permeability studies indicate that the lipophilic CD-C16 reside in the bilayer resulting in a more flexible bilayer membrane, while upon heating at temperatures near the transition from the gel phase to the liquid crystalline phase, the embedded lipophilic carbon dots most probably form carbon dot-rich subphases, which could provide a justification for the observed permeability increase and fast release. The available experimental data suggest that both electrostatic interactions and lipophilic interactions are contributing to the observed thermosensitivity, while work is needed to elucidate the underlining mechanism at the molecular level. In vitro cell viability studies confirmed their enhanced anticancer efficiency at this elevated temperature compared to non-temperature sensitive liposomes. In particular, alkylated carbon dots result in a fluid liposomal bilayer, significantly affecting its structural and thermodynamic properties, leading to fast DOX release. Accordingly, CD-C16 containing liposomes demonstrate enhanced cytotoxicity of encapsulated DOX under hyperthermia conditions (40 °C), but not at physiological temperature where its activity is significantly lower than that of free DOX. However, presumably due to this preferential uptake, they proved to be very cytotoxic against HEK293, a non-cancerous cell line even at 37 °C, being equally potent as free DOX, which could hamper their utility.

Alternatively, hydrophilic CD containing liposomes exert a moderate effect on membrane permeability, being more cytotoxic than simple liposomes and less cytotoxic than free DOX against DOX resistant cells at 40 °C, but are less toxic than free DOX against the non-cancerous HEK293 cell line at 37 °C. Therefore, CD containing liposomes could be preferable to CD-C16 containing liposomes in hyperthermia therapeutic schemes, since they are equally potent to free DOX at 40 °C and less potent at 37 °C. Although more work with a variety of various DOX resistant and DOX sensitive cell lines is clearly required, the results suggest that it is possible, through the incorporation of either hydrophilic or lipophilic carbon dots, to fine tune liposomal membrane permeability towards the development of effective drug delivery systems with cell internalization properties for hyperthermia-based applications.

## Figures and Tables

**Table 1:** The effect of the presence of carbon dots (CD) and palmitoyl-functionalized carbon dots (CD-C16) on the thermodynamic parameters (*T*_m_ and Δ*T*_½_) of the main lipid phase transition of DPPC:DSPE-PEG-based unilamellar liposome formulations, either empty or doxorubicin (DOX)-loaded, in RPMI, as obtained during the first and second heating run. The experimental errors of the *T*_m_ and Δ*T*_½_ values are ±0.2 °C. Data are representative of at least two independent experiments for each liposomal formulation.

Formulation	*T*_m_ (°C) First/Second Heating Run	Δ*T*_½_ (°C) First/Second Heating Run
DPPC:DSPE-PEG	41.1/41.2	2.1/2.1
DPPC:DSPE-PEG + DOX	40.6/41.1	1.4/1.4
DPPC:DSPE-PEG + CD	41.0_/_41.4	1.6/1.5
DPPC:DSPE-PEG + DOX + CD	40.9/41.2	1.4/1.3
DPPC:DSPE-PEG:CD-C16	42.0/42.3	2.4/2.4
DPPC:DSPE-PEG:CD-C16 + DOX	42.3/42.3	2.5/2.6

## Data Availability

The original contributions presented in this study are included in the article/Supplementary Material. Further inquiries can be directed to the corresponding author.

## Supplementary Materials

The following supporting information can be downloaded at: https://www.mdpi.com/article/10.3390/ph19050668/s1, Figure S1: ^1^H NMR (A) and ^13^C NMR (B) spectra of parent CD in D_2_O; Figure S2: ^1^H NMR spectrum of alkyl-functionalized CD in CDCl_3_ employing naphthalene as an internal standard; Figure S3: UV–Vis spectra of CD in water and of the alkylated CD-C16 derivative in ethanol.

## Abbreviations

The following abbreviations are used in this manuscript:

ATR	Attenuated Total Reflection
^13^C NMR	Carbon-13 Nuclear Magnetic Resonance Spectroscopy
C16	Palmitoyl alkyl chain
CD	Nitrogen-doped carbon dots
CD-C16	Palmitoyl-functionalized carbon dots
DAPI	4′,6′-Diamidino-2-phenylindole
DLS	Dynamic light scattering
DMSO	Dimethyl sulfoxide
DOX	Doxorubicin
DPBS	Dulbecco’s Phosphate-Buffered Saline
DPPC	Dipalmitoylglycerophosphocholine
DSC	Differential scanning calorimetry
DSPE-PEG	Distearoylglycerophosphoethanolamine-polyethylene glycol
EDTA	Ethylenediaminetetraacetic acid
EPR effect	Enhanced Permeability and Retention effect
FBS	Fetal bovine serum
FTIR	Fourier Transform Infrared Spectroscopy
^1^H NMR	Proton Nuclear Magnetic Resonance Spectroscopy
HEK293	Human embryonic kidney cell line
HEPES	4-(2-hydroxyethyl)-1-piperazineethanesulfonic acid
IPCA	1,2,3,5-tetrahydro-5-oxoimidazo[1,2-a]pyridine-7-carboxylic acid
LIP	PEGylated DPPC liposomes
Lip-CD	CD containing liposomes
Lip-CD16	CD-C16 containing liposomes
MDSC	Modulated differential scanning calorimetry
MTT	Thiazolyl blue tetrazolium bromide
PC3	Human prostate cancer cell line
PEG	Polyethylene glycol
RPMI	Roswell Park Memorial Institute medium
UV–vis	Ultraviolet-visible spectroscopy
