# Supplementary material for: Hydrophilic and Lipophilic Carbon Dots Impart Thermosensitivity to Doxorubicin Loaded Phospholipid Liposomes
Source: Pharmaceuticals (Basel). 2026 Apr 25;19(5):668. doi: 10.3390/ph19050668 (PMC13210328; doi:10.3390/ph19050668)
Supplement: Supplementary file 1 [file pharmaceuticals-19-00668-s001.zip › pharmaceuticals-4254101-supplementary.pdf]

Hydrophilic and Lipophilic Carbon Dots Impart Thermosensitivity to Doxorubicin Loaded Phospholipid Liposomes

Barbara Mavroidi, Kyriaki Marina Lyra, Zili Sideratou, and Dimitris Tsiourvas

Supplementary Material

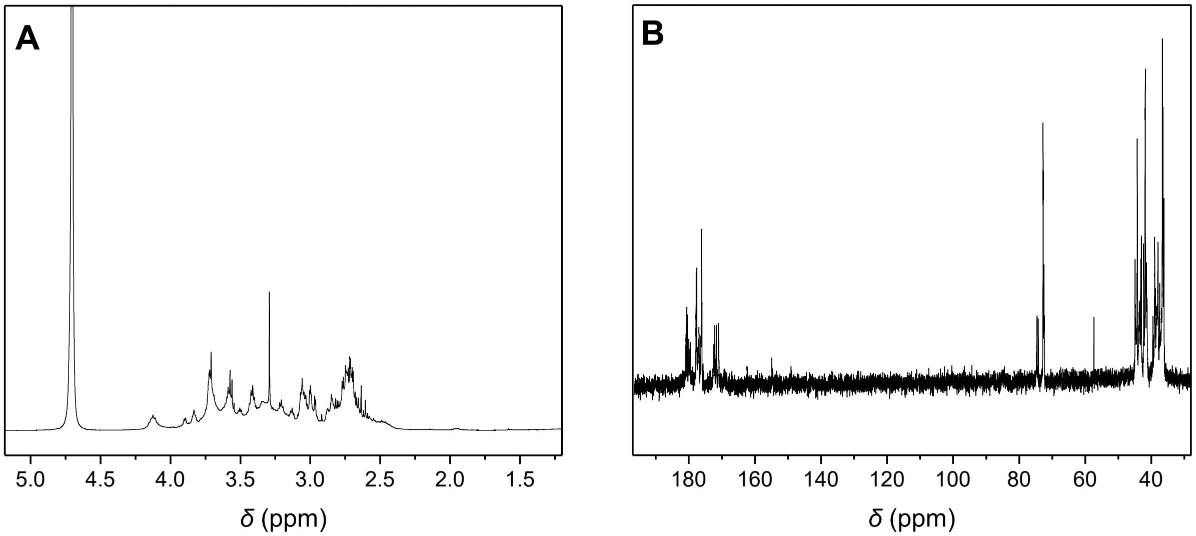

Figure S1. ^{1}H NMR (A) and ^{13}C NMR (B) spectra of parent CD in D_2O.

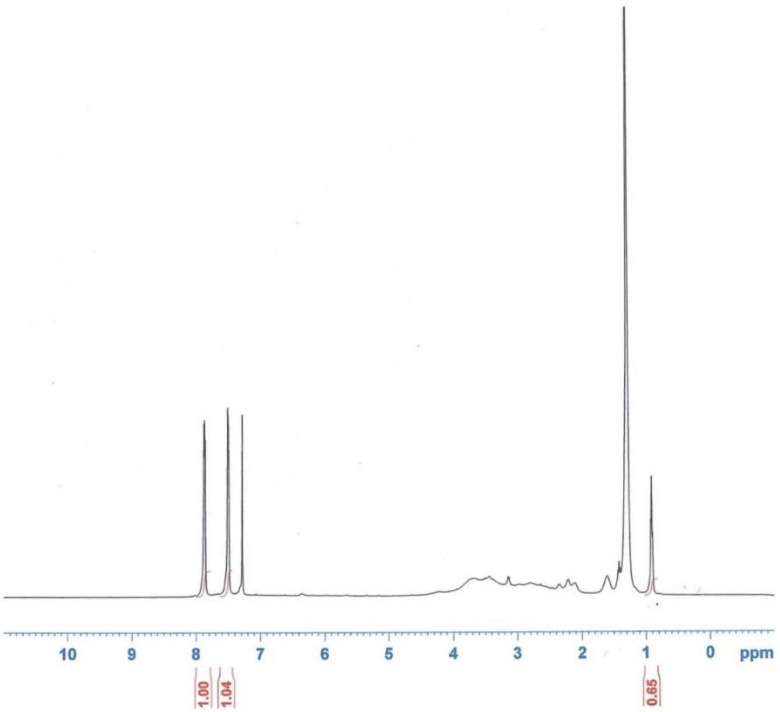

Figure S2. ^{1}H NMR spectrum of alkyl-functionalized CD in $CDCl_3$ employing naphthalene as an internal standard.

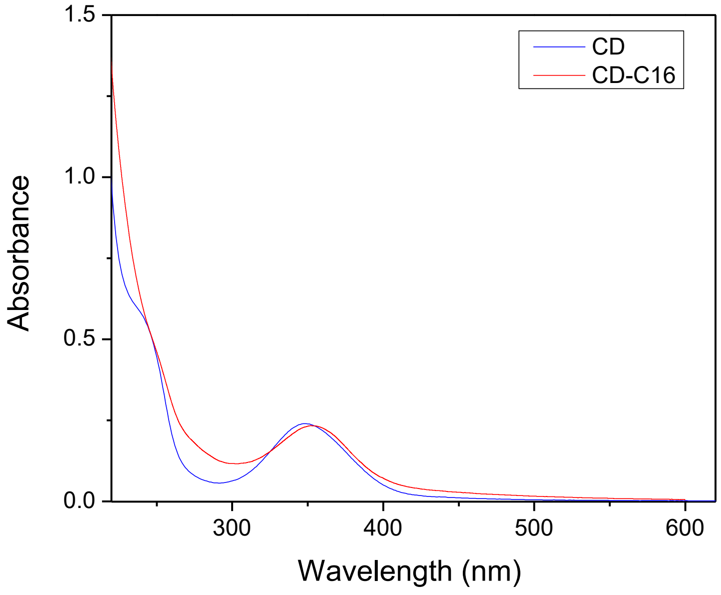

Figure S3. UV–Vis spectra of CD in water and of the alkylated CD-C16 derivative in ethanol.
